# An unusual presentation of a vaginal leiomyoma in a postmenopausal hysterectomised woman: a case report

**DOI:** 10.1186/1757-1626-2-6461

**Published:** 2009-03-10

**Authors:** Smitha V Nidhanee, Sachchidananda Maiti, Dillshad Shareef, Nigel Holland

**Affiliations:** 1 Department of Obstetrics and Gynaecology, Warrington and Halton Hospitals, NHS Foundation Trust, WA5 1QG, United Kingdom; 2 Department of Histopathology, Warrington and Halton Hospitals, NHS Foundation Trust, WA5 1QG,, United Kingdom

## Abstract

Leiomyomas are benign tumours commonly occurring in the uterine wall. They are rarely seen in the vaginal wall leading to pressure symptoms on urinary tract. Indentation of leiomyoma from anterior vaginal wall into the bladder is rare and hence we report one such case. A 55 year old Caucasian woman presented to urology clinic with recurrent Urinary tract infection and pressure symptoms. After the diagnosis of a vaginal mass, she was referred to Gynaecology clinic. During the excision of the vaginal mass its indentation into the bladder was noted. Histology report confirmed the diagnosis of benign leiomyoma.

## Introduction

The vagina is a rare site for leiomyoma, and they are usually located in the anterior wall and rarely from the lateral wall [1].They usually present as a mass in the vagina or as pressure symptoms on the urinary tract [2,3]. We report a case of leiomyoma arising from the anterior wall of vagina indenting laterally into the bladder wall, which lead to difficulties in its excision and subsequent bladder injury which was repaired eventually.

## Case presentation

A 55-year-old Caucasian woman presented to the urology clinic with complaints of sensation of pressure in suprapubic area with symptoms suggesting of possible cystitis for last twelve months. She had been treated for recurrent urinary tract infections in the past. She denied any dyspareunia and bowel dysfunction. She had two normal vaginal deliveries. She had undergone total abdominal hysterectomy 10 years ago and was on hormone replacement therapy for last five years.

**Figure 1:**
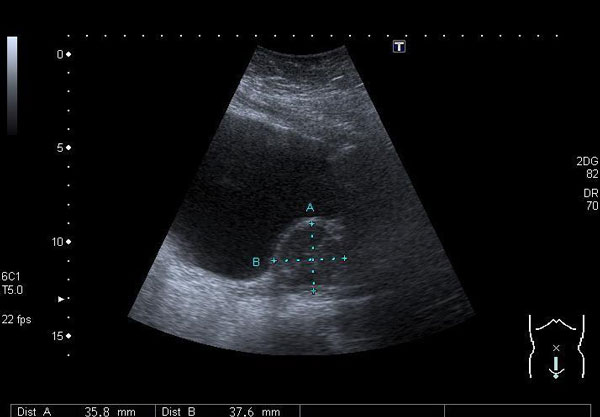
**Trans-abdominal scan**.

**Figure 2:**
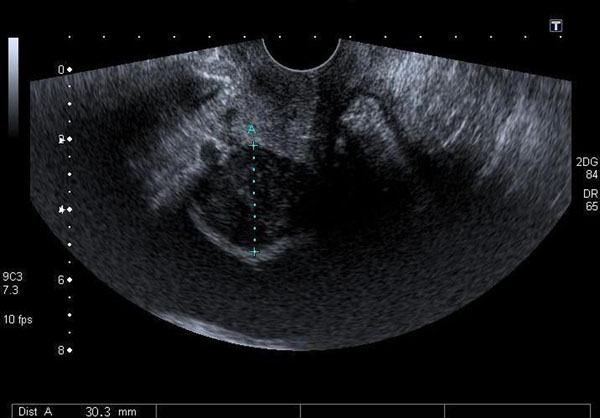
**Trans-vaginal scan**.

Trans-abdominal and trans-vaginal ultrasound scan revealed a 3 cm mixed echo solid mass indenting the urinary bladder in the distal anterior vaginal wall (Figure 1 &2). At this stage she was referred to the gynaecology team.

Gynaecological examination revealed that the vault was well supported with a small cystocele and rectocele and a 3-4 cm mobile cystic mass was palpable on the anterior vaginal wall about 1 cm from the vault. Subsequently, during examination under anaesthesia with a urologist in attendance, 3-4 cm solid mobile anterior vaginal mass was noted extending along the lateral vaginal wall. In an attempt to completely enucleate the mass, a small perforation was accidentally made in the bladder wall. The tumour was removed completely in piecemeal and sent for histopathology. The bladder was repaired in 2 layers by the urologist.

**Figure 3:**
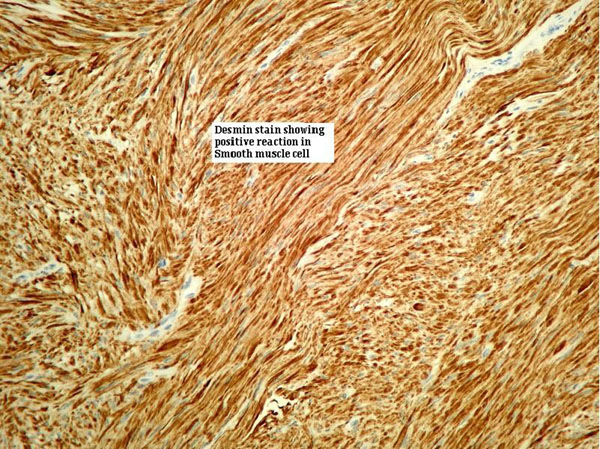
**Desmin stain showing positive reaction in tumour cells**.

She was discharged home on the third post operative day with catheter in situ and 10 days of antibiotics. Catheter was removed on the 10^th^ day following normal cystogram. Histology report showed smooth muscle tumour consisting of uniform smooth muscle cells without pleomorphism/mitotic activity thereby confirming benign vaginal leiomyoma (Figure 3,4 &5).

**Figure 4:**
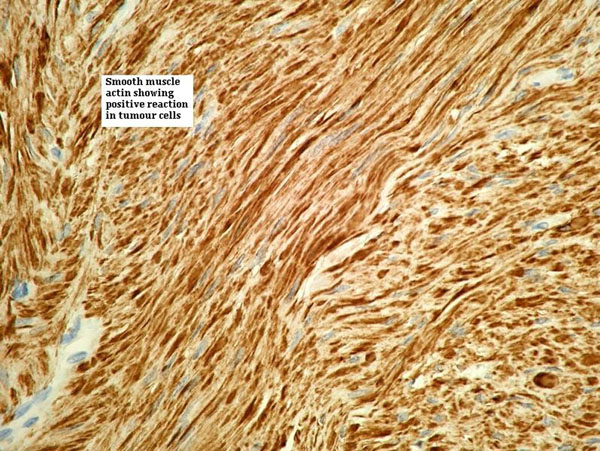
**Smooth muscle cells showing positive reaction to actin**.

**Figure 5:**
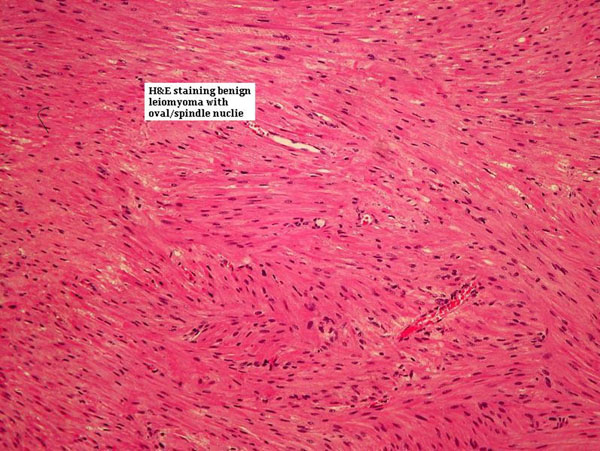
**Hematoxylin & Eosin stain showing benign leiomyoma with uniform oval/spindle nuclei**.

## Discussion

Over 300 cases of vaginal leiomyomas have been reported in the literature. They have varied clinical presentation, the most common being vaginal mass [4,5].

Vaginal myomas have been reported in patients from puberty to 71 years of age [6]. Average age of diagnosis tends to be in late 30s or early 40s. Vaginal myomas can be asymptomatic. When symptoms present, they commonly are related to the urinary tract and include voiding difficulties, dysuria and urinary frequency. The most common gynaecological complaint is dyspareunia. However, many patients only describe a bulging mass [6].

These lesions tend to be single and less than 5 cm in diameter which are usually localised and vary from solid to cystic in consistency. They are usually firm and can undergo degenerative changes and feel soft [7].The majority are found in the anterior wall of the vagina and only 10 to 20 % are found in the lateral wall.

They may be confused with a variety of benign vaginal tumours. A preoperative diagnosis is seldom made.

Though surgery through vaginal route is the treatment of choice, recurrence is of concern. Some authors have specifically recommended removal of the tumour enbloc to avoid any possible recurrence [7].

Indentation of Leiomyoma from the vaginal wall into the urinary bladder has not been reported in the literature. The purpose of presenting this case report is to emphasize that benign leiomyomas can present with just urinary symptoms. Though benign they can indent the bladder wall, which can render the surgical excision difficult with out damaging the bladder wall. We strongly recommend high index of suspicion of bladder injuries in such cases. We also recommend involving urologist during the surgery if required for the repair of the bladder.

## Conclusion

Benign vaginal leiomyomas indenting the bladder wall is challenging to excise. We conclude that patient should be consented for the possibility of bladder injury and its complications in all such cases.

## Consent

Written informed consent was obtained from the patient for publication of this case report and accompanying images. A copy of the written consent is available for review by the Editor-in-Chief of this journal.

## Competing interests

The authors declare that they do not have any competing interest.

## Authors' contribution

SN wrote the draft of the manuscript, performed the literature search and was involved in the care of the patient. SM supervised and finalised the manuscript. NH and DS contributed to her care and helped with the manuscript.
